# In-space manufacturing of optical lenses: Fluidic Shaping aboard the International Space Station

**DOI:** 10.1038/s41526-026-00629-6

**Published:** 2026-07-07

**Authors:** Omer Luria, Mor Elgarisi, Eytan Stibbe, Michael López-Alegría, Crissy Canerday, Alexey Razin, Sivan Perl, Valeri Frumkin, Jonathan Ericson, Khaled Gommed, Daniel Widerker, Israel Gabay, Ruslan Belikov, Edward Balaban, Moran Bercovici

**Affiliations:** 1 Faculty of Mechanical Engineering, Technion—Israel Institute of Technology, Haifa, Israel; 2 The Rakia Mission, Tel Aviv-Yafo, Israel; 3 Axiom Space, Houston, TX USA; 4 NASA Marshell Space Flight Center, Huntsville, AL USA; 5 NASA Ames Research Center, Moffett Field, CA USA; 6 Department of Materials, ETH Zürich, Zürich, Switzerland; 7 Present Address: Department of Mechanical Engineering, Boston University, Boston, MA USA

**Keywords:** Engineering, Materials science, Optics and photonics, Physics

## Abstract

In-space manufacturing technologies are considered vital for enabling advanced space missions and addressing logistical limitations of space exploration. While additive manufacturing has progressed rapidly, it still falls short of delivering the ultra-smooth surfaces required for optical elements. Fluidic Shaping is a novel method that harnesses surface tension under microgravity to form optical components with exceptionally smooth surfaces. This study demonstrates the feasibility and potential of Fluidic Shaping as a method for manufacturing optical components in space through two experiments performed aboard the International Space Station (ISS) during the Ax-1 mission. The first experiment involved fabricating centimeter-scale polymer lenses, solidifying them via ultraviolet (UV) curing, and analyzing the resultant optics upon their return to Earth. While sub-nanometric surface smoothness was achieved, some polymer lenses displayed unexpected thermo-chemical deformations, indicating complex polymerization dynamics unique to the microgravity environment. In the second experiment, a 172 mm diameter water lens was deployed, confirming Fluidic Shaping’s scalability and demonstrating basic optical functionality through image analysis. These experiments collectively underline the technique’s relevance for both small-scale optics and large-aperture applications. Our results highlight critical considerations for future research, including optimizing polymerization processes and refining liquid-handling methods to advance practical, in-space optical manufacturing capabilities.

## Introduction

As humanity continues to explore our solar system and beyond, technologies for in-space manufacturing will be indispensable, as long-duration missions must be self-sufficient. Additive manufacturing is of particular interest for space applications owing to its high versatility and has been repeatedly demonstrated in recent years onboard the ISS to fabricate a wide range of mechanical objects. Additive manufacturing also offers the potential to overcome launch constraints by fabricating objects larger than the launcher’s payload fairing limits^1–6^. Although additive manufacturing has notable advantages when relatively complex geometries are required, it cannot be applied to fabrication of macro-scale optics. Optical elements, such as lenses and mirrors, whether on a small or large scale, require extremely tight manufacturing tolerances and exceptionally low surface roughness (typically on the scale of nanometers) specifications, which are out of reach for all existing additive manufacturing technologies^7–9^.

A notable example of the need for optical manufacturing in space is corrective eyewear. During long-duration spaceflight, astronauts experience spaceflight-associated neuro-ocular syndrome (SANS), reporting decreased near vision of up to 1.5 diopters, potentially appearing as early as three weeks in microgravity^10–12^. As no known prevention or treatment for SANS exists, this syndrome poses a key risk for deep space exploration. Consequently, on extended missions, corrective eyewear may be essential to maintaining operational readiness and the quality of life of the crew. Given the unpredictable nature of vision decline, developing the capability to fabricate corrective eyewear in space may be very important. Another example where in-space manufacturing of optics may be required is in the creation of extremely large space telescopes. To achieve high resolution and light collection abilities, telescopes should ideally be as large as possible, yet launch limitations cap their size to just a few meters. As a direct consequence, direct imaging of exoplanets—widely regarded as the “holy grail” of modern astronomy—remains an elusive goal^13–16^.

Surprisingly, although optical components are at the forefront of many cutting-edge technologies, optical manufacturing has undergone very little innovation over the past centuries. The standard methods for the fabrication of lenses are based on mechanical processes such as grinding and polishing, or molding^17–20^. These processes are based on heavy machinery and require significant energy and water resources, which are inherently incompatible with space environments.

**Fig. 1:**
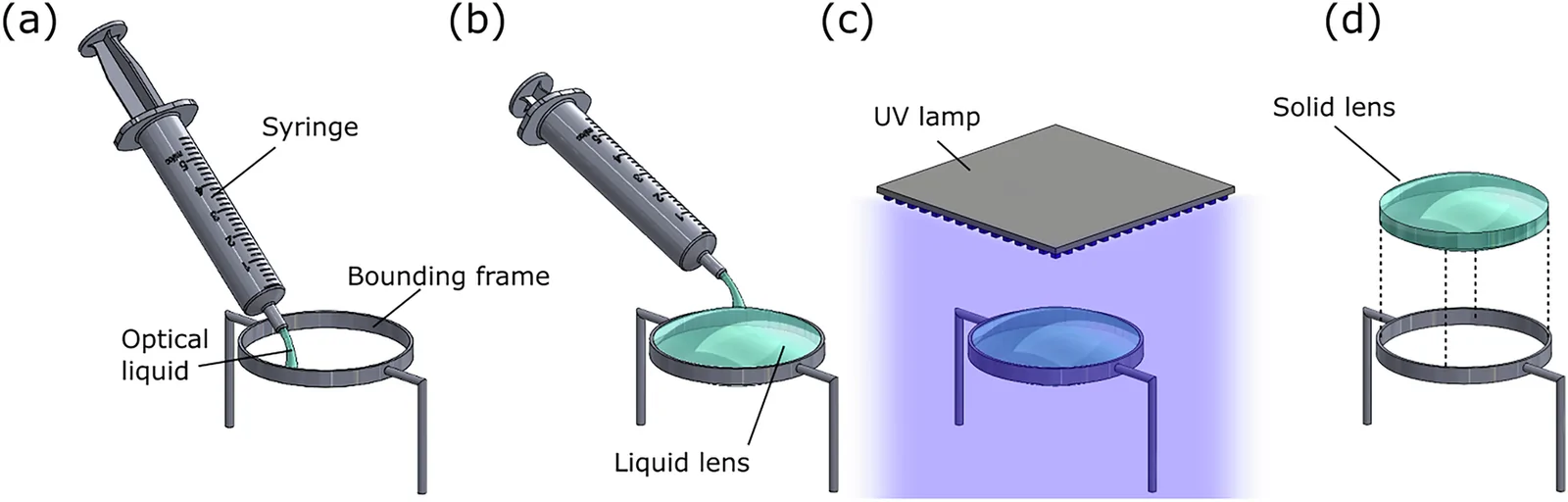
Fabrication of an optical lens using Fluidic Shaping under microgravity. **a** Optical liquid is injected into a circular bounding frame. **b** Once the frame is filled with liquid, the obtained minimum-energy state is a liquid lens with spherical free surfaces. **c**, **d** If the optical liquid is curable, it can be solidified (e.g., via UV light) to produce a solid lens.

Fluidic Shaping is a novel approach for the fabrication of optical components, such as lenses and mirrors, that leverages the natural surface tension of liquids under microgravity^21^. Under weightlessness, the shape of a liquid volume is prescribed solely by any solid boundaries it is in contact with, with its free surfaces characterized by constant mean curvatures. Injecting a liquid into a simple circular ring boundary will result in the shape of an optical lens with two spherical free surfaces. The radii of curvature of the surfaces (and thus the optical prescription of the lens) can then be changed simply by adding or removing liquid. The resulting surfaces are exceptionally smooth, naturally exhibiting sub-nanometric surface roughness. If the lens is made of a curable liquid (such as a photopolymer), it can be solidified into a rigid component. Figure 1 depicts the process of fabricating a lens using Fluidic Shaping. In the absence of body forces, the method is theoretically scale-invariant and can be used in the same fashion to create extremely large lenses, requiring only a larger bounding frame and larger liquid volumes. Frumkin and Bercovici^21^ and Elgarisi et al.^22^ have demonstrated the method on Earth by injecting the polymer into an immiscible immersion liquid of equal density to that of the polymer, thus achieving neutral buoyancy conditions. They have shown how different types of lenses (spherical, aspherical, and freeform) can be obtained by tuning the shape of the frame, the liquid lens volume, and the deviation from perfect neutral buoyancy.

Fluidic Shaping is inherently based on weightlessness conditions and is therefore directly applicable for in-space manufacturing of optics. Conceptually, applying Fluidic Shaping in space is simpler than doing so on Earth, as no immersion liquid is required. It is an excellent candidate for the creation of not only small components, such as ophthalmic lenses, but also of extremely large optics, such as those required for telescopes. The latter is manifested in the FLUTE project, which aims to overcome launch constraints and create a 50-meter liquid-based space telescope^23,24^. In previous work, we reported parabolic flight experiments in which we created liquid lenses under microgravity without the need for an immersion liquid^25^. We later extended this work to the creation of mirrors, using reflective liquids^26^. However, a fundamental challenge in parabolic flights is the acceleration profile, composed of repeating sequences of short (10-20 s) and noisy microgravity maneuvers. These conditions preclude lens curing, which requires an extended duration of precise microgravity. For this reason, in our parabolic flight experiments to date, all lenses and mirrors remained in liquid form.

In this paper, we report a step forward towards in-space manufacturing of optics by demonstrating the fabrication of lenses through two scientific experiments performed aboard the ISS. The experiments were performed in April 2022 as part of the Rakia Mission - the Israeli astronaut work plan aboard the ISS, as part of Axiom Space’s Ax-1 mission. In the first experiment, we created centimeter-scale lenses in microgravity and cured them using UV light. The solid lenses were then returned to Earth for testing and characterization. In the second experiment, we demonstrated the scalability of the method by deploying a 172 mm diameter lens made of water. We then performed image analysis on the video footage of the experiment to estimate the quality of the resulting lens.

## Methods

### Experimental hardware

**Fig. 2:**
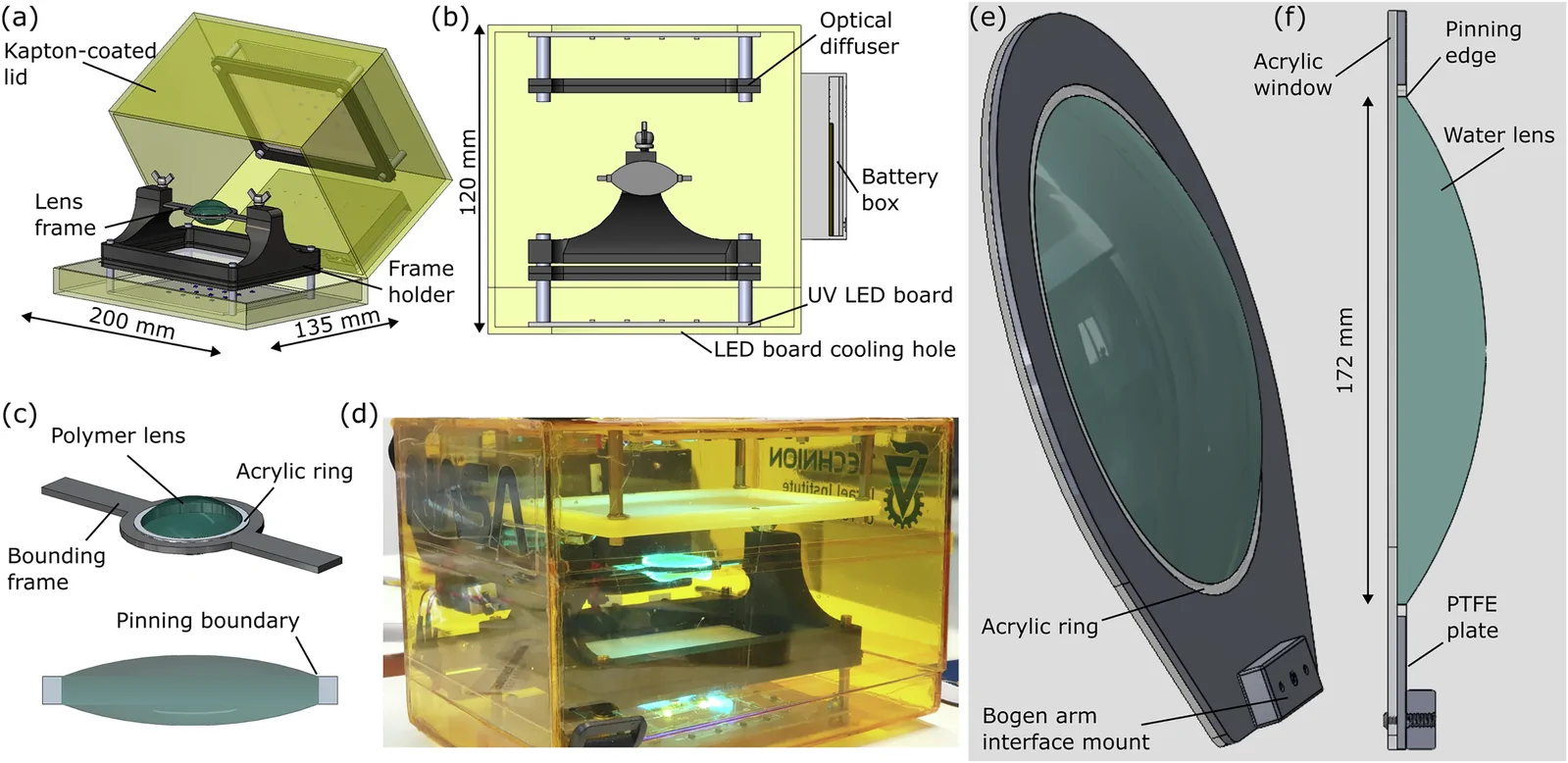
The hardware developed for the creation of solid and liquid lenses on the ISS. **a** Isometric and (**b**) cross-sectional views of the lens manufacturing chamber, which was used to shape and solidify polymer lenses. The chamber is composed of a base and a hinged lid, forming a closed chamber. Liquid polymer was injected into the lens frame, which was fixed at the center of the chamber by the frame holder. Once the lens was formed, the lid was closed, and UV LED boards at the base and on the lid of the chamber were turned on to polymerize the lens into a solid component. An optical diffuser was placed between the LEDs and the lens to provide uniform illumination. **c** The lens frame, into which the polymer is injected, includes an inner acrylic ring that provides pinning boundary conditions for the liquid to form the lens, and two elongated beams that secure it to the frame holder through two wing screws. **d** An image of the chamber, as taken on Earth prior to launch, with an empty bounding frame and the LEDs turned on. **e** Isometric and **f** cross-section views of the frame used to deploy a 172 mm diameter plano-spherical water lens. The frame was made of a flat acrylic window, attached to an annular PTFE plate with a thin acrylic ring fitted into it, which formed a shallow circular dish. An interface mount with a ¼” hole was connected to the bottom part of the frame for interfacing with standard Bogen arms available on the ISS.

Figure 2(a–d) presents the experimental hardware used to fabricate lenses under microgravity on the ISS. While Fluidic Shaping is highly versatile and compatible with a wide variety of liquids, the material’s behavior as it undergoes solidification critically affects the quality of the final component. Here, we opted for UV-curable polymers as they do not require bubble-free mixing and allow the astronaut to monitor and correct the injection before initiating the curing process. We thus designed the lens manufacturing chamber to serve both as a mechanical platform in which the liquid lenses were created and as a UV chamber, in which the lenses were subsequently solidified. Figure 2(a, b) shows isometric and cross-section CAD views of the chamber, which was composed of a base and a hinged lid. When closed, the lid prevented any airflow in or out of the chamber, to eliminate potential disturbances to the lens before curing. At the heart of the lens manufacturing chamber is a bounding frame with an internal acrylic ring, 30 mm in diameter, used as the pinning boundary for the Fluidic Shaping process. Under microgravity, upon the injection of liquid into the frame, a spherical lens is formed, as depicted in Fig. 1 and detailed in Section 1 of the Supplementary Information. To allow both convenient access during the injection and unobstructed UV exposure during curing, the bounding frame was supported in the center of the chamber by a fixed structure (the ‘frame holder’), such that the surfaces of the lens would be exposed from all directions. Two wing screws were used to affix the bounding frame to the frame holder, allowing to quickly placing and removing bounding frames from the holder. The frame holder was attached to the base of the chamber using four standoffs. Two UV light-emitting diode (LED) boards were attached to the base and lid, at equal distances below and above the bounding frame, and were covered with an optical diffuser made from a layer of parchment paper. The internal dimensions of the chamber and the distances between the bounding frame, LED boards, and diffusers were experimentally optimized to produce uniform UV illumination during the curing process.

Each UV LED board consisted of two arrays of 365 nm surface-mount technology LEDs: an array of 16 low-power LEDs (OCU-400 UB365, OSA Opto Light GmbH, Germany) powered at 15 mA, and an array of two high-power LEDs (ATDS3534UV365B, Kingbright, Taiwan) powered at 360 mA. The currents through each LED were regulated using two-terminal constant LED drivers (AL5809, Diodes Inc, USA). To provide sufficient heat dissipation from the LED boards, the back surface of the board was exposed to the environment through holes in the enclosure. Both LED boards were connected through a switch (not shown in the figure) to a box with eight AA alkaline batteries connected in series. The switch allowed the astronaut to separately turn on either the low- or high-power arrays. To adhere to the ISS safety regulations, the length of the battery power line was minimized by connecting the switch to the LEDs via a relay box, which was placed right next to the battery. In addition, a limit switch was placed on the lid of the chamber to prevent the LEDs from working while the lid is open, and all the external surfaces of the chamber were coated with Kapton tape to prevent any exposure to UV, even when the lid is closed.

Three different photopolymer types were used as lens materials: a polyurethan-based resin (TJ-3704A, also sold under the brand name ‘Vida Rosa’, Dongguan Tianxingjian Electronic Technology Co., Ltd, Dongguan, China) and two optical adhesives (NOA 61 and NOA 63, Norland Products Inc., NJ, USA). These materials were selected based on our previous experience in lens fabrication in the lab. Each material was filled into a Luer-lock syringe, which was capped and wrapped in Kapton tape to keep the polymer protected from ambient UV during handling and transport. The Kapton-coated syringes were then packed in UV-opaque bags and, in contrast to the rest of the equipment, which was delivered to the ISS several months in advance on a resupply mission, the polymers were launched just prior to the experiment together with the Ax-1 crew.

The UV curing experiment was performed inside the Life Science Glovebox (LSG), located in the Japanese *Kibō* module of the ISS. The LSG kept the experiment in negative pressure and thus protected the crew from possible outgassing or spillage of the liquid polymers. During the experiment, the astronaut connected the lens manufacturing chamber to the workbench of the LSG using four Velcro pads, which were placed under the base of the chamber. To create the lens, the astronaut opened the lid, placed an empty lens frame into the frame holder, and injected liquid polymer into the acrylic ring to create the lens. Once the liquid stabilized, the astronaut closed the lid, turned on the low-power UV arrays for 4 min, and then turned on the high-power UV array for another 4 min to finish the curing. After the curing, the astronaut opened the lid, removed the solidified lens, stowed it in a dedicated box, and repeated the process with a new bounding frame. The experiment was monitored and recorded using three cameras: a GoPro camera providing side view, an over-the-shoulder Canon XF705 camcorder, and a Nikon D-6 outside of the LSG.

Figure 2(e, f) shows isometric and cross-section CAD views of the frame used for the second experiment, where a large-scale (172 mm in diameter) liquid lens was deployed in the open cabin. The only liquid that could be used safely outside the glovebox is water, and thus, this experiment was based entirely on the standard water supply of the ISS. The main challenge with deploying large-scale lenses is creating a stable and continuous liquid connection across the entire aperture while maintaining precise pinning boundary conditions. For this reason, we capped the frame with a flat acrylic window so that the liquid would wet the entire aperture, resulting in a plano-spherical lens. The acrylic window was covered with an annular PTFE plate, into which a thin acrylic ring with a sharp edge was fitted. The acrylic window and ring formed a shallow circular dish, which provided the boundary conditions (through adhesion to the window surface and pinning to the ring edge), while the PTFE was used as a hydrophobic layer to prevent water from advancing and wetting the entire plate. At one end of the frame, an aluminum mount with a ¼” threaded hole was installed to provide an interface to a Bogen arm—a standard ISS attachment boom.

### Astronaut training

**Fig. 3:**
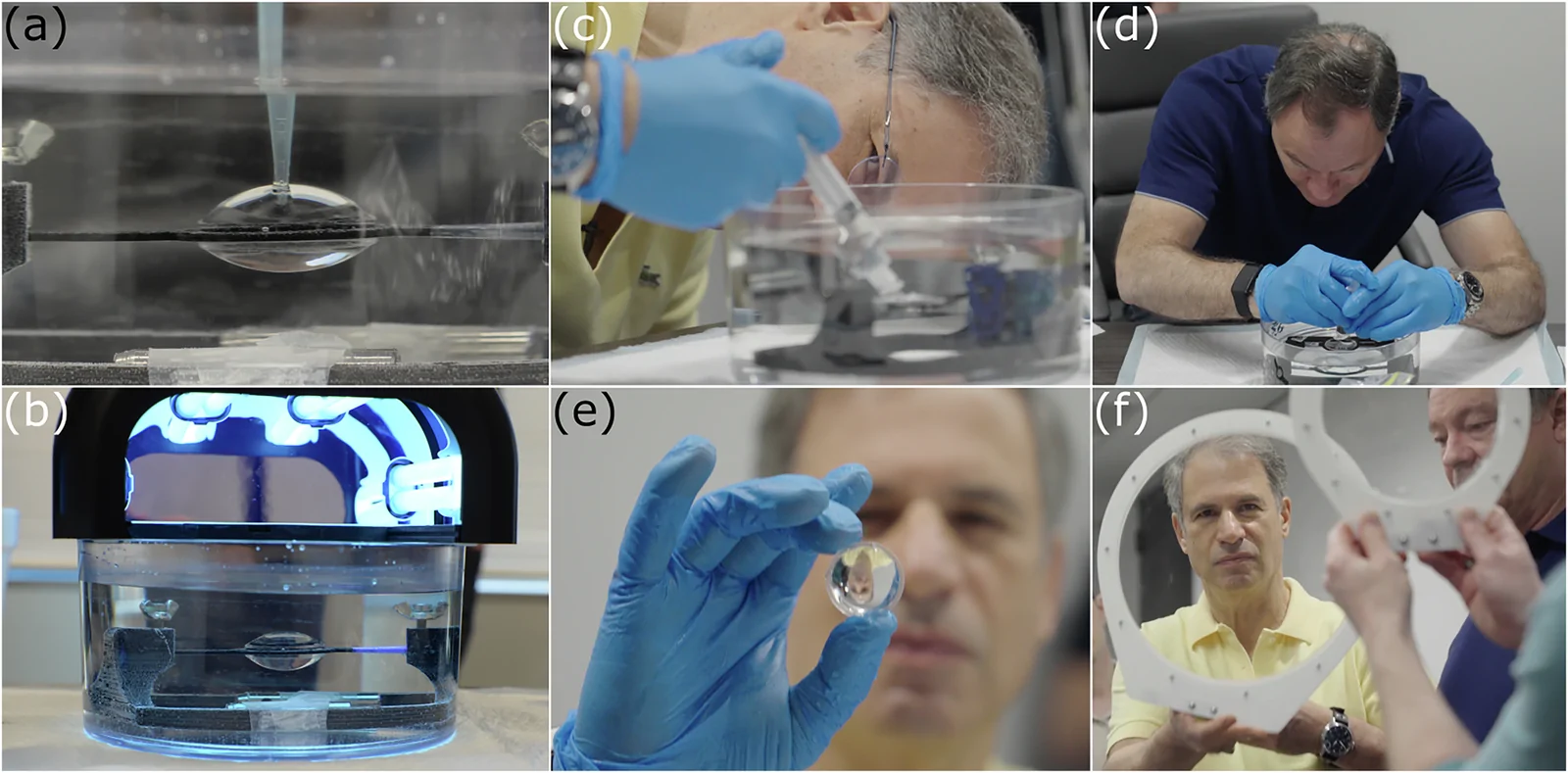
Astronaut training sessions prior to Ax-1 launch. **a**, **b** The training setup, based on neutral buoyancy to simulate microgravity. A replica of the frame holder was placed at the bottom of a tank full of an immersion liquid, and liquid polymer was injected into the bounding frame to create the lens. A UV lamp was used to cure the lenses. **c**, **d** Astronauts Eytan Stibbe and Michael López-Alegría training in creating lenses under neutral buoyancy. **e** One of the solidified lenses was produced during the training session after removing it from the immersion liquid. **f** The astronauts examining the frames intended for deploying large-scale lenses. The authors have received written consent from all the individuals appearing in the images.

Before the Ax-1 mission launched, several training sessions were held to get both of Ax-1 astronauts who were in charge of the experiment familiar with creating lenses using Fluidic Shaping, as well as with operating the specific experimental hardware. Figure 3 shows select images from our training sessions in Axiom Space’s facility in Houston, Texas. Figure 3(a, b) shows the setup that was used to train the astronauts on injecting and curing polymer lenses. The training setup consisted of a lens frame and frame holder identical to the ones used in the flight unit. To simulate microgravity conditions, the frame was submerged in a mixture of water and glycerol at a density equal to that of the polymer, such that neutral buoyancy was achieved. A standard commercial off-the-shelf mercury lamp (DR-301C, Melody Susie, US) was placed on top of the liquid immersion container and was used as a UV source to cure the lenses. Figure 3(c, d) shows the astronauts injecting a liquid photopolymer into the lens frame under neutral buoyancy. The astronauts repeated the experiment several times and practiced achieving proper pinning on the frame’s edges, removing bubbles, and curing. Figure 3(e) shows astronaut Eytan Stibbe holding such a solidified lens, immediately after taking it out of the immersion liquid. Figure 3(f) shows both astronauts examining the frames intended for the deployment of large-scale water lenses.

An additional training session was held in the simulator of the ISS LSG at NASA Johnson Space Center (JSC) in Houston, Texas. The objective was to familiarize the astronauts with the LSG, as well as plan the exact positioning of the experimental hardware inside the LSG.

## Results

### UV curing of polymer lenses

**Fig. 4:**
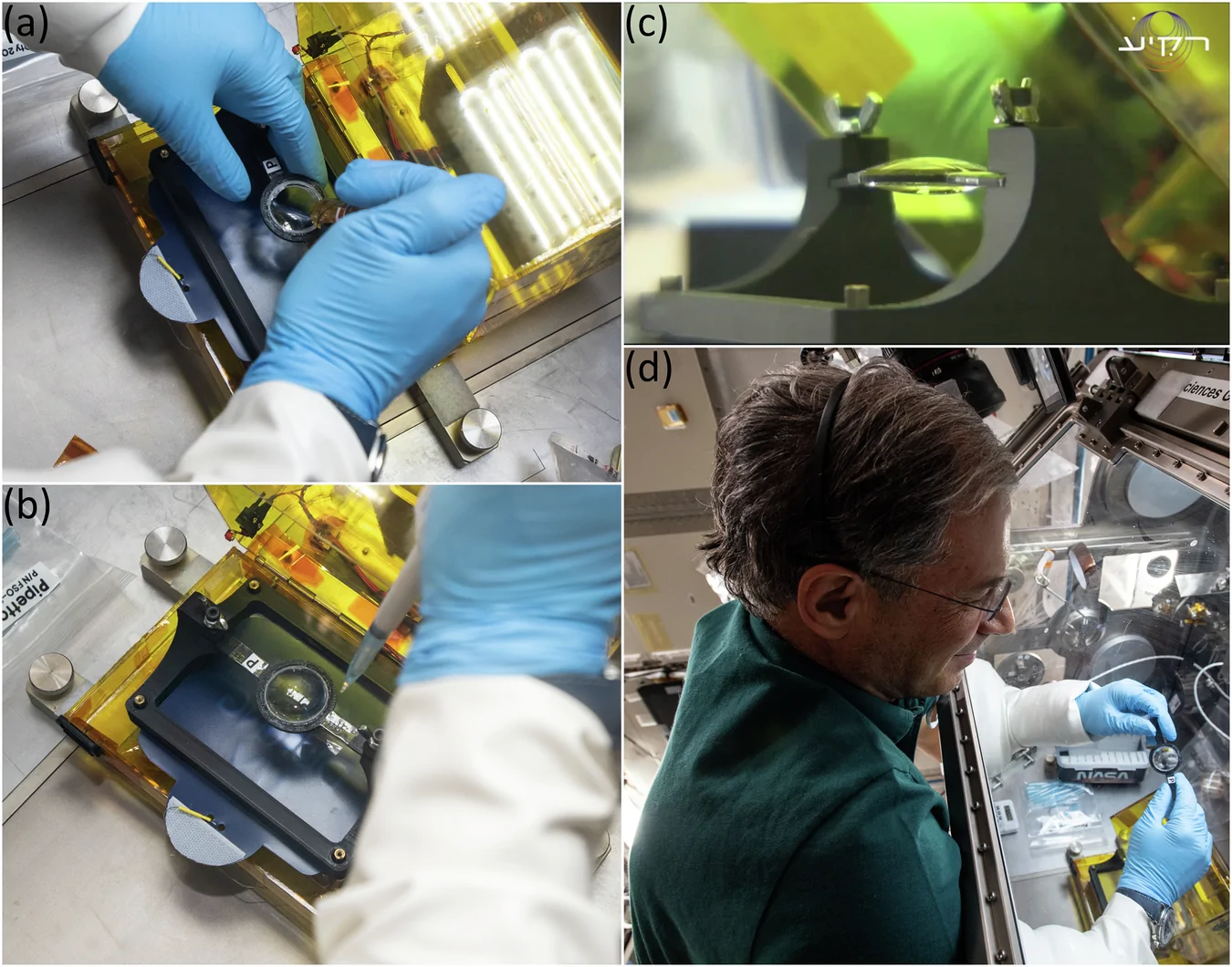
In-space manufacturing of polymer lenses using Fluidic Shaping in the LSG. **a** Filling the frame with liquid polymer under microgravity, in the same fashion as was done in the training (Fig. 3), but without the need for an immersion liquid. **b** Once the frame was filled, a liquid lens was obtained. **c** A side view showing the lens before curing. **d** The astronaut holding the solidified lens after curing. Photographed by NASA astronaut Kayla Barron. The authors have received written consent from all the individuals appearing in the images.

The UV curing experiment was conducted in the LSG over the course of three hours, during which a total of four polymer lenses were fabricated. Two lenses were made with TJ-3704A, one lens was made with NOA 61, and the last one was made with NOA 63. Figure 4(a–c) presents select images from the experiment, showing filling of the frame to create the lens, and a side view of the liquid lens prior to curing. Figure 4(d) shows the astronaut investigating the first solid lens after curing. All four lenses were packed by the astronaut in a dedicated box and returned to Earth in a subsequent cargo resupply mission for inspection in the lab.

The first two lenses were fabricated from TJ-3704A. After curing the first one, the astronaut visually inspected the solidified lens and noticed many small dimples across the surface of the lens—a phenomenon which we have never observed when fabricating lenses from TJ-3704A in neutral buoyancy^22,27^. After observing the same phenomenon in the second lens, we decided to fabricate the third and fourth lenses from NOA 61 and 63, respectively. These did not exhibit such defects.

**Fig. 5:**
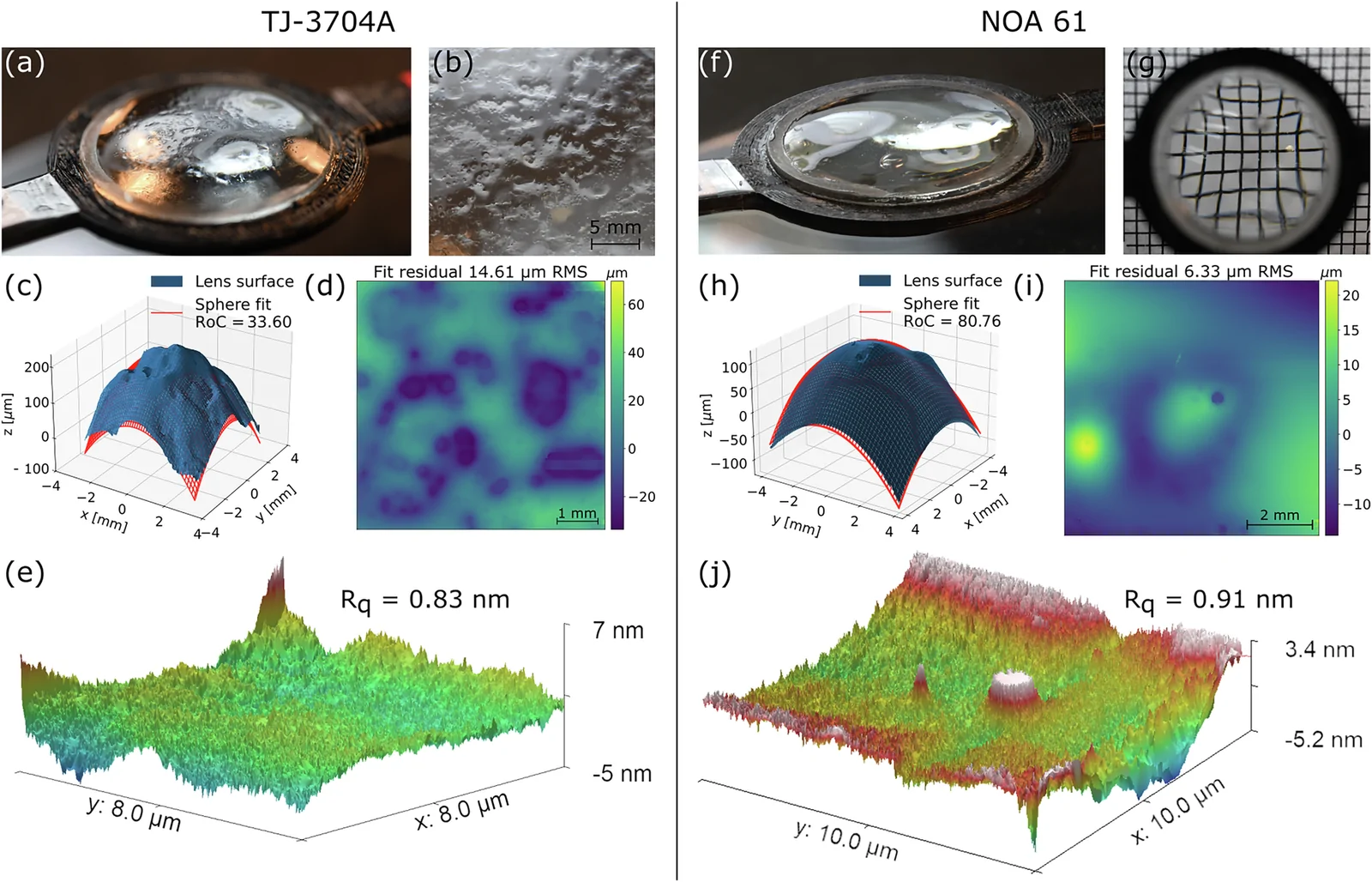
Lab characterization of the cured TJ-3704A and NOA61 lenses. **a**, **f** Photographs of the lens fabricated in space. **b** Close-up view showing the dimples on the surface of the Vida Rosa lens. **g** A photograph of a grid pattern taken through the NOA61 lens to demonstrate its refractive ability. **c**, **h** Topography maps around the center of the top surfaces of the lenses, showing the surfaces and their best-fit spherical caps. **d**, **i** Deviation of the surfaces from perfect spheres, obtained by subtracting the sphere fits from the measured surfaces. **e**, **j** AFM mapping of the surface, showing sub-nm surface roughness values for both lenses.

Figure 5 shows lab characterization results of two of the lenses—a TJ-3704A lens and a NOA 61 lens, both with a diameter of 30 mm. Figure 5(a) shows a photograph of a TJ-3704A lens, and Fig. 5(b) shows a magnified view of the dimples on the surface. The dimples are in the range of 0.1–1 mm in diameter, and their appearance suggests bubble formation on the surface of the lens, which might be a result of some form of boiling of the polymer components during the curing process, either due to excess temperature rise and/or as a product of the reaction itself. This is supported by Video 2 of the Supplementary Information, which shows fumes escaping the chamber as the astronaut opened the chamber’s lid after polymerization. Figure 5(c, d) shows topographical mapping of the central part of the lens surface, performed using a digital holographic microscope (DHM R1000 with 20x objective, LynceeTec, Switzerland). Due to the large sag of the lens, we were able to scan only part of the aperture with moderate slopes, as steeper slopes could not be captured by the instrument. Although the dimples clearly distort the surface, the base shape nominally forms a convex spherical cap, with a radius of curvature of 33.6 mm and an asphericity of 14.61 µm RMS. Figure 5(e) shows a nanoscale topographical mapping performed with an atomic force microscope (AFM, Asylum MFP3D, Olympus AC240 cantilever operated in tapping mode). The dimples do not appear to affect the nanoscale topography, and the surface roughness is within sub-nm RMS values (*R*_q_ = 0.83 nm, over a 8 × 8 μm area), consistent with previous results obtained via Fluidic Shaping^21,22^. Fig. 5(f–j) shows a similar characterization for the NOA 61 lens. Except for several bubbles close to the free surface, the lens is free of local imperfections. Figure 5(g) shows an image of a grid pattern taken through the lens, showing the expected pincushion distortion due to its bi-convex morphology. As shown in Fig. 5(h–i), topographical mapping confirms that the NOA 61 lens is much closer to a perfect spherical topography than the TJ-3704A lens, with asphericity of 6.33 µm RMS. AFM measurements also confirm sub-nm surface roughness for the NOA 61 lens (R_q_ = 0.91 nm, over a 10×10 μm area).

In our setup, we deliberately chose to enclose the lens in a chamber during curing to prevent potential disturbances due to vent airflow inside the LSG. As microgravity eliminates natural convection, our enclosed chamber created a highly insulated thermal environment, where heat could dissipate from the lens only by conduction through the surrounding air and by radiation. These conditions differed markedly from those under which we optimized our system on Earth, and from the conditions in which we fabricated lenses out of the same TJ-3704A polymer under neutral buoyancy in previous works^22,27^. Although we had tested equally thick polymer layers without noticeable deformation, it is likely that natural convection in those settings was sufficient to remove excess heat during curing. In contrast to the TJ-3704A lenses, the NOA lenses showed no such defects, indicating that their polymerization chemistry generates less heat or follows different thermal dynamics.

### Deployment of a 172 mm diameter liquid lens

**Fig. 6:**
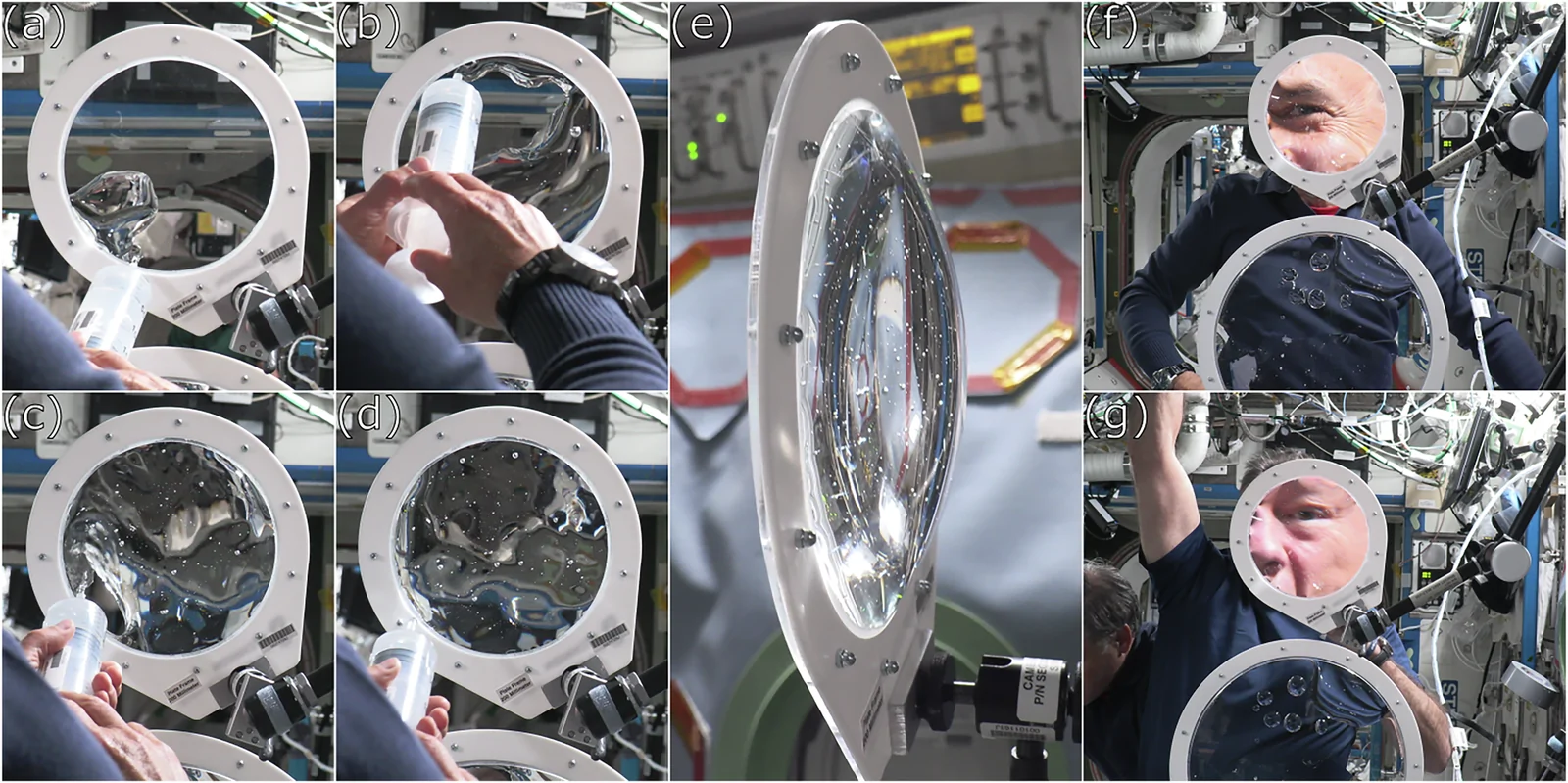
Deployment of a 172 mm diameter liquid lens. **a**–**c** Filling of the frame in a spiral motion to properly pin the water to the acrylic edge and adhere to the acrylic window. **d** Once the frame was filled, a plano-convex lens was formed. **e** A side view showing the stable lens after the liquid has settled. **f**, **g** Astronauts Eytan Stibble and Michael López-Alegría demonstrating the magnifying effect of the lens. Photographed by Ax-1 astronauts Michael López-Alegría and Eytan Stibbe. The authors have received written consent from all the individuals appearing in the images.

Figure 6 shows the deployment of a 172 mm diameter liquid lens in the US *Destiny* module of the ISS. The size of the setup required this experiment to be performed in the open cabin. A camera (XF705 camcorder, Canon, Japan) was positioned on a Bogen arm, facing the starboard wall. In front of the camera, the frame was fixed to another Bogen arm, such that the frame was parallel to the wall, sufficiently far from the air vents to prevent disturbances due to air flow. A 200 ml syringe was filled with water, taken directly from the drinking system of the station. The astronaut deployed the lens by injecting water directly onto the frame’s acrylic window, and another astronaut refilled the syringe each time it depleted. Figure 6(a-d) shows the process of filling the frame to create the lens. During the injection, the astronaut moved the syringe in a spiral motion to properly pin the liquid to the edge of the acrylic ring. Once all the liquid fronts met and a continuous volume filled the frame, a spherical plano-convex lens was formed, as shown in Fig. 6(e). This technique required a relatively large initial volume of water, resulting in a liquid lens with an exaggerated curvature. The manual process also resulted in air bubbles trapped within the lens. Since the lens remained liquid (and thus self-healing), the astronaut could aspirate the liquid with a syringe to remove the bubbles and, at the same time, reduce the lens’ curvature. Once the lens was brought to the desired curvature, its magnification was demonstrated by standing near its focal plane, as demonstrated in Fig. 6(f, g). The astronaut further aspirated the liquid volume several times, with apparent real-time changes to the image magnification, as shown in the Supplementary Information Video 3. After the experiment had finished, the astronaut aspirated the water back into the syringe and the fed it back to the water system of the ISS for recycling. Remaining water was absorbed with a towel.

The experiment was originally intended solely as a qualitative demonstration. Nevertheless, the video footage can also be used to provide quantitative estimates for the lenses’ performance. A simple and universal quantity for such an estimation is the modulation transfer function (MTF), which represents the resolving ability of an optical system, i.e., its ability to transfer spatial frequencies from the object space to the image space^28,29^. Among the many different ways to measure the MTF, the most natural one to apply in our case is the *slanted-edge method*, which was standardized as part of ISO 12233^28,30–33^. We refer the reader to the Standard for a full explanation of the method, and here provide only a brief description of its implementation. In short, a sharp edge target (a one-dimensional step function in intensity) is placed at the object plane, and the image of that edge is then used to extract the inherent frequency response of the system. The sharp edge is purposely positioned at a slant angle to the pixel grid of the sensor, to allow for super-sampling of the edge’s position, to within sub-pixel precision.

A general difficulty in interpreting MTF measurements is that the frequency response is a property of an entire optical system rather than of a specific element within it. In our case, the contrast of the obtained image is a function of the sharpness of the target, the design and quality of both the liquid lens and the camera lens, the performance of the image sensor (including any digital manipulation performed by the camera processor), and the exact configuration in which all the components are positioned with respect to one another. Therefore, to associate the performance metrics of the system with an individual element, its contribution to the measured response must be dominant. In the case of this experiment, we made the assumption that — given the high quality of the rest of the elements of the optical chain — the liquid lens is the dominant contributor to the degradation in image sharpness. To validate this assumption, we set up a numerical Zemax model wherein the liquid lens is modeled as a plano-spherical lens, and the rest of the optical system and sensor are ideal. Agreement between the measurements and simulation would cross-validate the two and confirm that the liquid lens is indeed the central source of aberrations.

**Fig. 7:**
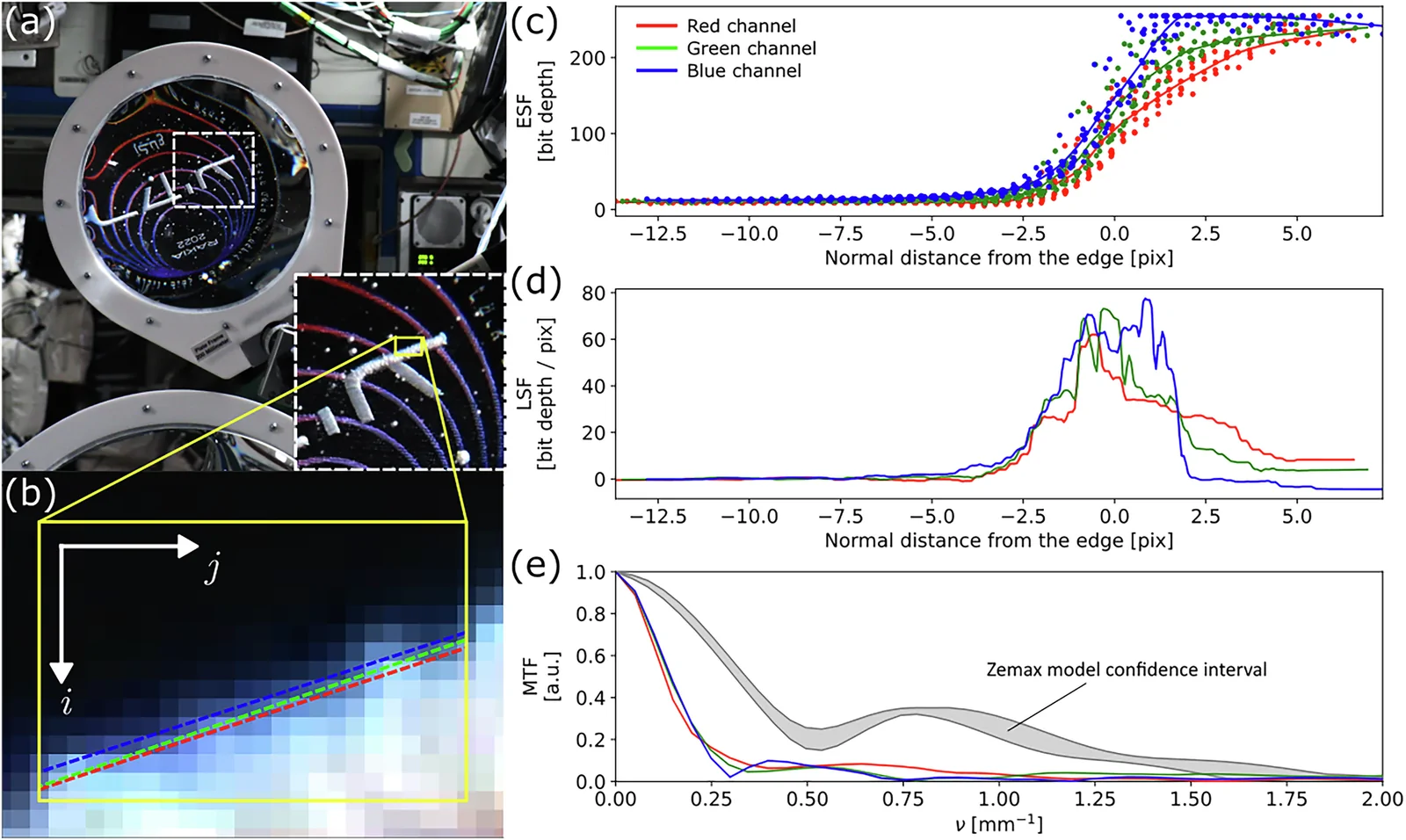
Estimation of the resolving ability of the liquid lens. **a** A close-up view of the magnified target after gamma decoding. The inset shows a zoomed-in view of the analysis area. **b** The rectangular region of interest in which the MTF analysis is performed. **c** The super-sampled edge-spread functions (ESFs) within the region of interest, and smooth curves produced via local regression. **d** The line spread functions (LSFs) are computed by taking the first derivatives of the ESFs. **e** The modulation transfer functions (MTFs), which are the moduli of the Fourier transform of the LSFs, as functions of spatial frequency in the object space. The gray area represents the simulated polychromatic sagittal MTF in the Zemax model, for different combinations of our setup parameters, accounting for their uncertainty. The different colors in all graphs correspond to different channels (red, green, and blue) in the sRGB image. Photograph in (**a**) by Ax-1 astronaut Eytan Stibbe.

Figure 7(a) shows the selected image for the analysis, where the Rakia mission patch is positioned behind the lens and produces a magnified image. The white text on the dark background creates a slanted edge appropriate for the MTF analysis. As the experiment was not originally designed to be quantitative, the positions of the different elements, along with the focal length and aperture settings of the camera, were not precisely defined, nor were they recorded during the experiment. Moreover, some of these parameters, such as the camera position and focus setting, have changed during the experiment. For this reason, we must retrieve these parameters via triangulation, based on known dimensions of the elements in the ISS module. We describe this triangulation process in detail in Section 2 of the Supplementary Information.

Once the optical setup parameters are retrieved, we are able to move on to the MTF analysis on the image. The image, taken from a video, is sRGB and so the analysis is performed on each color channel (red, green and blue) separately. We first perform gamma decoding to remove the nonlinear corrections performed by the camera processor and obtain an image with intensity values that linearly depend on the incident radiant flux. The opto-electronic transfer function (specified by the manufacturer) follows the ITU-R BT.709 standard^34–36^, with the inverse transformation given explicitly for each pixel separately as

1 $$I=\left\{\begin{array}{ll}V/4.5 & {\rm{if}}\,\,V < 0.081\\ {\left(\frac{V+0.099}{1.099}\right)}^{1/0.45} & {\rm{if}}\,\,V\ge 0.081\end{array}\right.$$

where V is the encoded color intensity as saved by the camera, and I is the decoded intensity. The image shown in Fig. 7(a) is the gamma-decoded version of the original image (available in Figure S2 in the Supplementary Information). The inset shows a zoomed-in view of the relevant region on the target, where i, j represent the indices for the row and column, in the pixel array, respectively. The yellow rectangle in Fig. 7(b) is the region of interest (ROI) on the edge, in which the analysis is performed. For each pixel column j and color channel $k\in \{{\rm{Red}},{\rm{Green}},{\rm{Blue}}\}$, we take a central difference of the intensity profile,${I|}_{j,k}(i)$, and define the maximum of its absolute value as the position of the edge, corresponding to the inflection point of the intensity profile. We then fit a linear curve ${i}_{E}(j,k)=m(k)\cdot j+n(k)$ to those points, defining the edge line, as indicated by the dashed lines in Fig. 7(b). Next, an edge spread function ${\rm{ESF}}[s(i,j,k),k]$ is constructed as the intensity of each pixel within the ROI as a function of its normal distance s from the edge,

2 $$s(i,j,k)=(i-{i}_{E}(j,k))\cdot \,\sin (\arctan (-m(k))=({i}_{E}(j,k)-i)\frac{m(k)}{{m}^{2}(k)+1}$$

The slant angle provides sub-pixel precision, as pixels from different columns in the ROI are projected together onto a single intensity profile without overlapping. This results in a “super-sampled” ESF with a sample resolution higher than the original image. To remove high-frequency noise from the ESF, we perform local regression with the LOWESS algorithm^37,38^, and use this smoothed version in the rest of the analysis. Figure 7(c) shows the raw and smoothed ESF as point markers and continuous curves, respectively. The ESF is essentially the step response of the system, and therefore its first spatial derivative, shown in Fig. 7(d) is the one-dimensional impulse response of the system and also called the line spread function (LSF),

3 $${\rm{LSF}}(s,k)=\frac{\partial ({\rm{ESF}}(s,k))}{\partial s}$$

The MTF is then the modulus of the Fourier transform of the LSF,

4 $${\rm{MTF}}(\nu )=\left|{\int_{\min (s)}^{\max (s)}}({\rm{LSF}}(s)\cdot w(s)\cdot {e}^{-i2\pi \nu s}){ds}\right|$$

where ν is the spatial frequency along the s direction and w(s) is the Tukey window function with a shape parameter of 1.0^31^.

Figure 7(e) shows the MTF extracted from the image, in comparison with the polychromatic sagittal MTFs simulated in Zemax (gray area). The gray area indicates the range of MTF values arising from uncertainty in the retrieval of the setup parameters. Further details are provided in Section 3 of the Supplementary Information. We compare the empirical results to polychromatic theoretical curves and not to monochromatic ones, since the color channels in the camera have wide bandwidths and spectrally overlap^39^. We do not know, however, the exact responsivity of the camera sensor, and so we use a simple average between 486 nm, 588 nm, and 656 nm, as typically done in optical designs intended for the entire visible region.

We observe a reduction in modulation compared to the Zemax model, which we associate mainly with lossy video compression. Although we have used the highest-quality video format available to us (MXF format with 10 bits per channel), it is still compressed. It is impossible to precisely predict how compression affected the MTF; however, in the vast majority of cases, it leads to loss of contrast and thus to a reduction in the MTF^40^. Aside from compression, the reduction in the MTF could also be a result of optical aberrations. The most likely source of aberrations is liquid deformations on the free surface of the water lens. The microgravity levels on the ISS are known to be very precise^41,42^, yet disturbances from mechanical vibrations that coupled to the lens and may have not fully settled, as well as the air ventilation system, could be significant sources of aberration. Such aberrations can also result from imprecise boundary conditions, which lead to non-spherical geometry. Although we took extra care in the design to create ideal pinning conditions, non-perfect pinning can be observed in several points around the perimeter of the lens. This can be a result of dust particles and/or oil residues (e.g., from fingerprints). Elgarisi et al.^22^ have shown that high-frequency boundary distortion exponentially decays as a function of the radial distance from the boundary. However, low frequencies are attenuated much more slowly and may still produce non-negligible aberrations throughout the entire aperture of the lens. Another possible source of optical aberrations in the liquid lens is scattering by air bubbles trapped within the water lens during deployment.

## Discussion

In this work, we report on the first implementation of Fluidic Shaping for in-space manufacturing of optical components, in a set of experiments performed on board the ISS. Using a lens manufacturing chamber and a large-scale deployment frame designed specifically for these experiments, we demonstrated the creation and UV curing of centimeter-scale polymer lenses and the deployment and characterization of a large liquid water lens.

While lab characterization of our UV-cured lenses confirms sub-nanometric surface roughness, similar to that obtained by Fluidic Shaping in neutral buoyancy, we observed unexpected dimples on the lenses made from the TJ-3704A polymer. In contrast, the NOA lenses showed no such dimples. In follow-up work, we showed that the origin of these defects its localized boiling due to heat generated during polymerization^43^. These findings point to an important direction for future research: investigating the kinetics and thermodynamics of polymerization under microgravity, with particular attention to heat transfer mechanisms, curing environments, and material-specific behavior.

Our large-scale liquid optics experiment demonstrated both the scalability and the material-versatility nature of Fluidic Shaping. However, the manual process of spreading the liquid across a large aperture, while also achieving proper wetting and pinning, proved challenging. This highlights the need for more advanced liquid deployment strategies suitable for large apertures with minimal bubble formation. Although Fluidic Shaping is scale-invariant in the ideal, capillary-dominated theoretical case, perfect weightlessness is impossible to achieve in practical systems, and thus some degree of unintentional asphericity is unavoidable. These considerations are key for future liquid-based space observatories such as FLUTE^44,45^. While the experiment was primarily intended as a qualitative demonstration, we were able to extract first-order quantitative performance metrics through image analysis. To achieve higher precision and consistent optical quality, future systems will require integrated, in-situ characterization tools capable of monitoring fluidic optical component formation in real time, similar to what we have implemented in our parabolic flight setups^25,26^.

In both experiments, astronaut involvement provided valuable flexibility in orbit, enabling real-time response to unexpected developments. However, manual liquid handling also introduced operational challenges, such as trapped air bubbles and inconsistent wetting. Future experiments should transition toward more automated systems, with crew members serving in a supervisory capacity.

## Supplementary information

Supplementary Information

## Data Availability

Videos of the water lens experiment and the raw AFM data are publicly available in the following repository: https://doi.org/10.5281/zenodo.16931251.

## Code availability

The Python code used to analyze images from the water lens video is provided in the above repository.
