# Supplementary material for: In-space manufacturing of optical lenses: Fluidic Shaping aboard the International Space Station
Source: NPJ Microgravity. 2026 Jul 7;12:89. doi: 10.1038/s41526-026-00629-6 (PMC13714310; doi:10.1038/s41526-026-00629-6)
Supplement: Supplementary file 1 [file 41526_2026_629_MOESM1_ESM.pdf]

# Supplementary Information

# In-space manufacturing of optical lenses: Fluidic Shaping aboard the International Space Station

**Omer Luria [1], Mor Elgarisi [1], Eytan Stibbe [2], Michael López-Alegría [3], Crissy Canerday [4], Alexey Razin [1], Sivan Perl [1], Valeri Frumkin [1,§], Jonathan Ericson [1], Khaled Gommed [1], Daniel Widerker [1], Israel Gabay [1], Ruslan Belikov [5], Jay Bookbinder [5], Edward Balaban [5,*] and Moran Bercovici [1,*]**

[1] Faculty of Mechanical Engineering, Technion – Israel Institute of Technology, Haifa, Israel

[2] The Rakia Mission, 3 Shadal St, Tel Aviv, Israel

[3] Axiom Space, 1290 Hercules Ave, Houston, TX, USA

[4] NASA Marshell Space Flight Center, Huntsville, AL, USA

[5] NASA Ames Research Center, Moffett Blvd., Moffett Field, CA, USA

[§] Current affiliation: Department of Mathematics, Massachusetts Institute of Technology, Cambridge, MA, USA

[*] Corresponding authors: edward.balaban@nasa.gov (E.B), and mberco@technion.ac.il (M.B)

**The following additional files are provided in the Supplementary data repository[1] at https://doi.org/10.5281/zenodo.16931251**

1. **Video1.mp4:** A video showing main cuts of the UV curing experiment.
2. **Video2.mp4:** A video describing the procedure of the UV curing experiments.
3. **Video3.mp4:** A video showing main cuts of the large-scale liquid optics experiment.
4. **P1_VR_top_6000um_20x.tiff:** Surface profilometry measurements of the VidaRosa lens
5. **P2_NOA_TOP_7500um_2_2025-03-20.tiff:** Surface profilometry measurements of the NOA lens
6. **DHM_tiff_reader.zip:** A Python code used to analyze and plot the surface profilometry data.
7. **AFM_data.zip:** AFM data files of the VidaRosa and NOA lenses. Requires Gwyddion to read.
8. **MTF_analysis.zip:** The image and Python code used for the MTF analysis of the large liquid lens.
9. **Zemax_models.zip:** The Zemax model used to estimate the theoretical MTF of the large liquid lens.

## 1. Theoretical Background

The derivation of the Fluidic Shaping theory under buoyancy conditions is presented in detail in previous work by Frumkin et al.[2] and by Elgarisi et al.[3]. We here provide a summary of the theory, with a focus on the specific conditions relevant for this work. Consider a volume of optical liquid of density $\rho$ that is injected into a circular cylinder of radius $R_0$ and vertical thickness $H$, submerged within another immiscible liquid of density $\rho_{\mathrm{im}}$ (called the 'immersion liquid'), as shown in Figure S1(a). In the case where the optical liquid is pinned to the edges of a circular boundary, the energy of the system can be expressed as

$$\Pi = \gamma \int_0^{2\pi}\int_0^{R_0}\left[\sqrt{1+(h_r^{\,t})^2}+\sqrt{1+(h_r^{\,b})^2}-\frac{(\rho-\rho_{\mathrm{im}})g}{2\gamma}\left((h^t)^2-(h^b)^2\right)+\frac{\psi}{\gamma}\left(h^t+h^b\right)\right] r\,dr\,d\theta\,, \tag{S1}$$

where the top and bottom liquid interfaces $h^t$ and $h^b$ are assumed to be rotationally symmetric and must satisfy the volumetric constraint,

$$V_{lens} = \pi R_0^{\,2} H + 2\pi \int_0^{R_0}\left(h^t+h^b\right) r\,dr\,. \tag{S2}$$

In equation (S1), the first term in the integral represents the surface energy of the liquid-fluid interfaces, the second represents the gravitational potential energy that includes Earth's gravity and the hydrostatic buoyancy force, and the third represents a constraint on the finite volume of the injected liquid. $r$ and $\theta$ are the radial and azimuthal coordinates, $\gamma$ is the interfacial energy between the two liquids, $g$ is Earth's gravity, and $\psi$ is a Lagrange multiplier. The minimum energy state, where $\Pi$ has a global minimum, can be expressed by the Euler-Lagrange equations,

$$\left(\frac{(\rho-\rho_{\mathrm{im}})g}{\gamma}h^t-\frac{\psi}{\gamma}\right)+\frac{r h^t_{\,rr}+h^t_{\,r}+\left(h^{\,t}_r\right)^3}{r\left((1+\left(h^t_{\,r}\right)^2\right)^{3/2}}=0 \tag{S3}$$

and

$$\left(-\frac{(\rho-\rho_{\mathrm{im}})g}{\gamma}h^b-\frac{\psi}{\gamma}\right)+\frac{r h^b_{\,rr}+h^b_{\,r}+\left(h^{\,b}_r\right)^3}{r\left((1+\left(h^b_{\,r}\right)^2\right)^{3/2}}=0\,. \tag{S4}$$

For the case of either perfectly neutral buoyancy ($\rho-\rho_{\mathrm{im}}=0$, Figure S1(a)) or under microgravity conditions ( $g=0$, Figure S1(b)), the potential energy term vanishes and the shapes of each of the interfaces can be described by the following differential equation[4,5]

$$\frac{r h_{rr}+h_r+\left(h_r\right)^3}{r\left(1+\left(h_r\right)^2\right)^{3/2}}=\frac{\psi}{\gamma}\,, \tag{S5}$$

where $h = h^t = h^b$ due to symmetry. As the left-hand-side in equation (S5) represents the mean curvature of the surface, the conclusion is that under microgravity conditions either surface has a constant mean curvature. Since the boundary is circular, the result is that both surfaces are spherical caps, with a radius of curvature of $\frac{\gamma}{\psi}$, which is determined solely by the volume of injected optical liquid. For a transparent optical liquid with refractive index $n$, this yields a symmetric spherical lens (bi-convex or bi-concave) with a paraxial focal length according to the lens makers formula[6]

$$f = \left[ (n-1)\left( 2\frac{\psi}{\gamma} + \left(\frac{\psi}{\gamma}\right)^2 \frac{(n-1)(H + 2h|_{r=0})}{n} \right) \right]^{-1}. \tag{S6}$$

So far, the analysis has been focused on a cylindrical boundary with two open ends, forming two free surfaces. A special case can be considered in which the cylinder is capped on one end by a flat window, forming a dish. This constrains the capped liquid surface to be flat, i.e., $h^b = 0$ (assuming the bottom one is capped, without loss of generality) as shown in Figure S1(c). The resulting lens is then plano-spherical with a focal length of

$$f = \left[ (n-1)\left( \frac{\tilde{\lambda}}{\gamma} + \left(\frac{\tilde{\lambda}}{\gamma}\right)^2 \frac{(n-1)(H + h|_{r=0})}{n} \right) \right]^{-1}. \tag{S7}$$

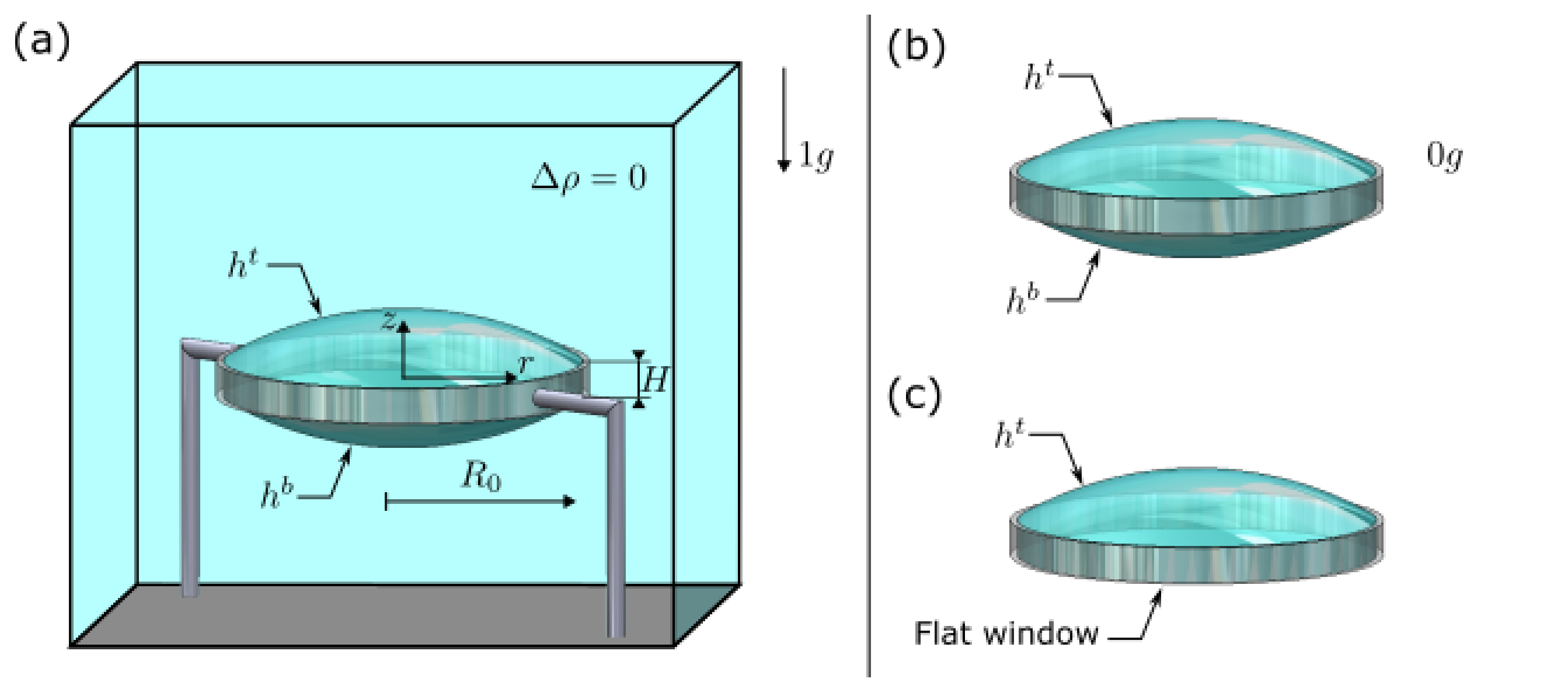


**Figure S1. Fluidic Shaping in neutral buoyancy and under microgravity.** (a) In neutral buoyancy, gravity effects are counteracted by matching the density of the immersion liquid to that of the optical liquid. When injecting an optical liquid into a hollow cylinder, the result is a bi-spherical lens. (b) Under microgravity, the same effect is achieved without the need for an immersion liquid. (c) The same principles hold when the cylinder is capped on one end with a flat window, only now there is a single free surface, which results in a plano-spherical lens.

## 2. Retrieval of the large-scale liquid optics setup parameters

Figure S2(b) shows a schematic drawing of the experiment setup. The camera lens (with an effective focal length of $f_c$) and the liquid lens are positioned at a distance $l_c$ from each other, and at a lateral distance $l_d$ between their optical axes. The target is positioned at a distance $l_t$ behind the lens and decentered by a distance $l_p$ from its optical axis. The longitudinal distance between the liquid lens and the starboard wall is denoted by $l_w$, and is estimated to be 2029 mm from CAD drawings of the Destiny module, provided by NASA.

The first step of the analysis is to estimate the distance $l_c$ and the focal length of the camera $f_c$. Consider an arbitrary point $W$ on the starboard wall, which is imaged on the camera sensor. A ray transmitted from that point intersects the plane of the liquid lens at point $C$ and continues toward the camera lens. For the estimation of $l_c$ and $f_c$, we neglect the physical size of the camera lens because it is negligible compared to the longitudinal distances between the camera, liquid lens, and starboard wall, which are several meters each. We denote the scale of the image (in mm/pixel) on the planes of the starboard wall and the liquid lens as $p_w$ and $p_c$, respectively. We use features small enough (in terms of angular size in the image) and close enough to the optical axis of the camera, so that both parallax and distortion errors are negligible, and thus consider $p_w, p_c$ to be uniform on each of the planes. On the lens' plane, the lens frame is the reference feature, with a diameter of 172 mm. On the starboard wall plane, the internal ISS intercom device (Audio Terminal Unit, or ATU) serves as the reference feature, with a height of 10.5" as shown in Figure S2(d) (data kindly provided by NASA). The lengths $l_c$ and $l_w$ can be related to the lateral distances $x_w$ and $x_c$ between the camera optical axis and the points $W$ and $C$, respectively, by

$$l_c = \frac{1}{x_w / x_c - 1} l_w . \tag{S8}$$

Since points $W$ and $C$ are imaged on the exact same point on the sensor, $x_w$ and $x_c$ have the same size in pixels, and so the ratio $x_w / x_c$ is simply the ratio of the scale of the image in the two planes,

$$\frac{x_c}{x_w} = \frac{p_c}{p_w} . \tag{S9}$$

Substituting equation (S9) into equation (S8), and using the known geometrical parameters, we obtain $l_c \approx 1186 \text{ mm}$. The focal length $f_c$ is determined using geometric optics by equating the observed and theoretical

magnification. The magnification $M$ is defined as the ratio of image to object size, and can be estimated from the image as $M = \frac{d_s}{N_c p_c}$, where $N_c$ is the width of the image in pixels and $d_s$ is the width of the sensor in mm. For a thin lens, the theoretical magnification is given by $M = \frac{f_c}{l_c - f_c}$[6]. Equating the two expressions and solving for $f_c$ yields

$$f_c = \frac{d_s}{p_c N_c + d_s} l_c. \tag{S10}$$

For $N_c = 3840\ \mathrm{pix}$ and $d_s = 13.2\ \mathrm{mm}$ (provided in the manufacturer's specification sheet), we obtain an estimate of $f_c \approx 19\ \mathrm{mm}$, which is within the range of the zoom camera lens (8.3 – 124.5 mm).[7] In contrast to the focal length, the size of the camera lens aperture cannot be directly extracted from the image. For this reason, we can only bound the entrance pupil size between the two extreme values of $f/2.8$ and $f/27$, according to the camera specifications.[7]

The remaining parameters required to fully retrieve the optical setup are the longitudinal and lateral distances of the patch relative to the axis of the liquid lens, $l_t$ and $l_p$. We determine their values using Zemax, by constructing the optical model and optimizing the distances to produce the same image size observed in Figure S2(a). To that end, we model the liquid lens as a plano-convex lens with a refractive index of 1.33[8] and a radius of curvature of 144 mm, determined by a best-fit spherical cap as shown in Figure S2(c). Since the camera is proprietary and the internal structure of its compound lens is not known, we model it as a single paraxial surface with a focal length of $f_c$, and a diameter equal to the entrance pupil in the two possible scenarios (either $f_c/2.8$ or $f_c/27$). The full parameters of the resulting Zemax model are provided in the next section.

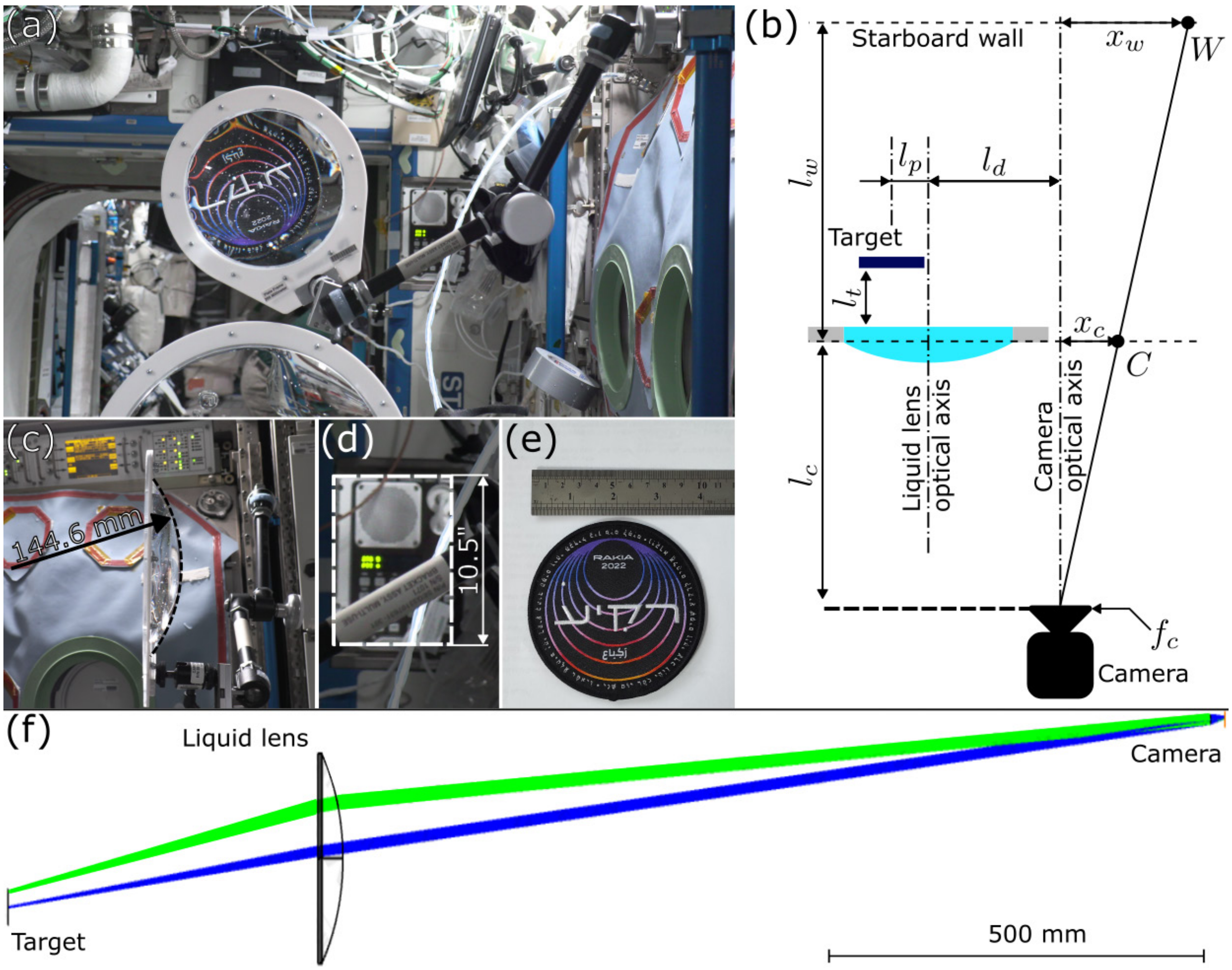


**Figure S2. Retrieval of the experimental parameters through triangulation on the image.** (a) The chosen image from the video footage, showing the Rakia mission patch magnified by the liquid lens. The image was chosen such that the details on the mission patch are as sharp as possible and oriented so that the slant angle is suitable for quantitative MTF analysis. (b) A schematic drawing of the experimental setup (not to scale). The target was placed behind the liquid lens, and the magnified image was captured by the camera. Using the dimensions of objects observed in the image and the distance between the planes of the liquid lens and the starboard wall, the distance between the camera and the liquid lens, as well as the effective focal length of the camera lens, are estimated via triangulation. (c) A side view of the liquid lens, showing curve fitting of the optical surface to a spherical cap, providing an estimate for its radius of curvature. (d) The internal intercom system of the ISS, used to estimate the scale of the image at the starboard wall plane. (e) A photograph of the Rakia mission patch, with a ruler for scale. (f) After the distance between the camera and liquid lens is obtained, the optical train is modelled in Zemax, with $l_t$ and $l_p$ as free parameters, which are varied until the patch image matches the magnification and decenter in the experiment. The camera lens was modelled as a single paraxial surface with a diameter equal to the entrance pupil. Photographed by Ax-1 astronaut Eytan Stibbe.

## 3. Optical modelling of the large-scale liquid optics setup in Zemax OpticStudio

Figure S3 shows the Zemax optical model used in analyzing the large-scale water lens ISS setup. The water lens was modeled as an assembly of three surfaces – one acrylic surface (corresponding to the window) and two water surfaces (corresponding to the water volume enclosed within the frame and to the water forming the spherical cap).

To provide our model with certainty intervals, we take into account the uncertainty in the parameters we extract from the triangulation analysis. We assume typical uncertainties of ±0.1 mm in the height of the ATU and the diameter of the liquid lens (nominal dimensions of 172.0 mm and 266.7 mm), and ±1 mm in $l_w$ (nominal dimension of 2029 mm). We then solve equations (S8)-(S10) for all permutations on a specified parameter grid, and use these results to obtain a range of possible configurations for the Zemax model. All configurations (including the nominal cases) constitute the uncertainty range presented in Figure 7 of the manuscript, and are consolidated in two .zmx files, as part of the .zip archive in the Supplementary Information.

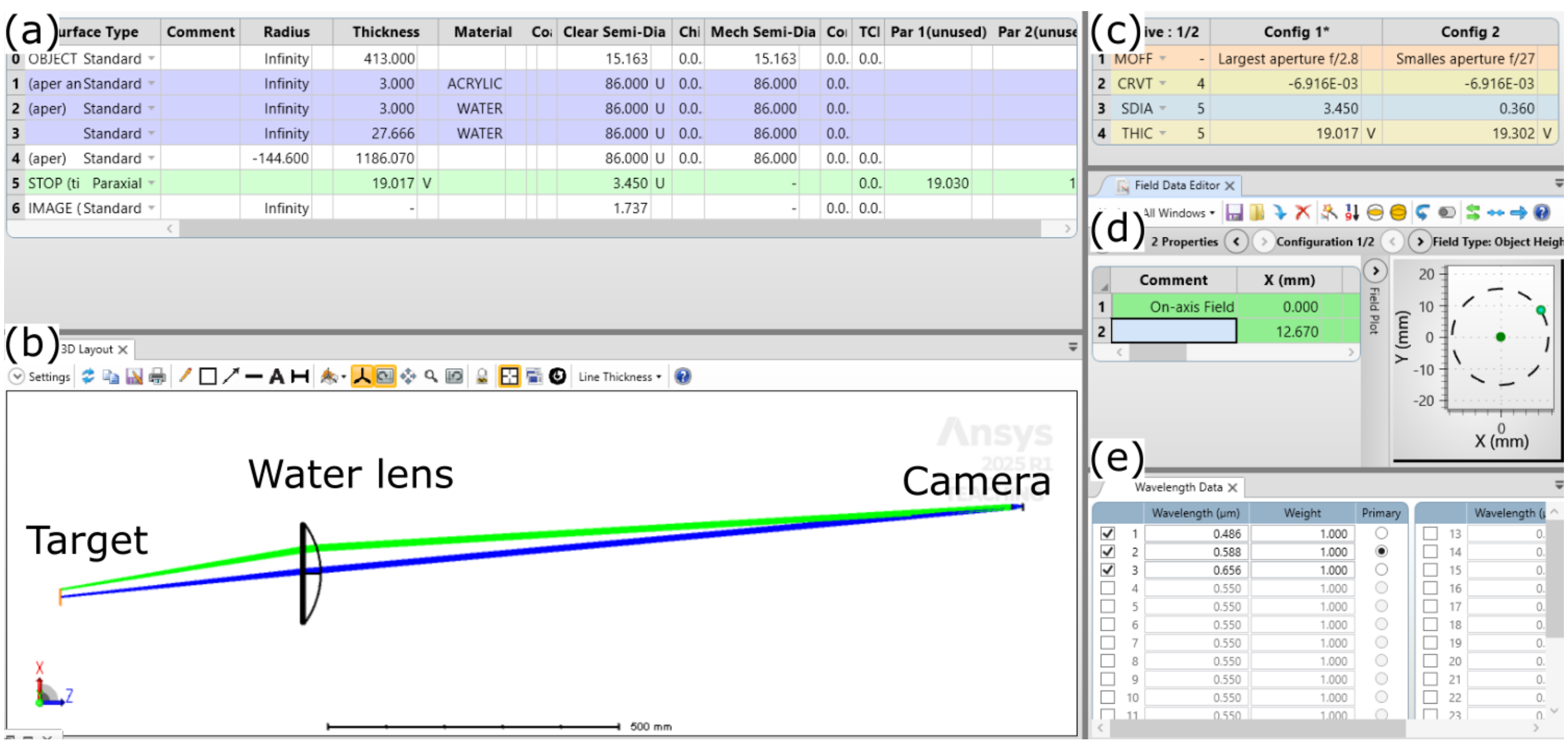


**Figure S3. Zemax model of the large-scale liquid optics setup. (a)** The lens prescription table, showing the converged solution. The liquid lens is modeled as an assembly of three surfaces – an acrylic surface accounting for the window, a flat water surface accounting for the enclosed volume in the dish, and a spherical water surface. The optical properties of the acrylic and water were taken from the Zemax materials library. (b) A ray diagram of the model, showing the target, water lens, and camera, to scale. (c) The configuration table, showing the different values for different camera aperture settings (f/2.8 and f/27). (d) The field data editor, showing the converged values for the lateral and longitudinal object position $l_t, l_p$. (e) The wavelength data editor, showing the wavelengths taken for the RGB MTF analysis.

## References


1. Luria, O. *et al.* Supplementary data for 'In-space manufacturing of optical lenses: Fluidic Shaping aboard the International Space Station'. Zenodo (2025).

2. Frumkin, V. & Bercovici, M. Fluidic shaping of optical components. *Flow* **1**, E2 (2021).

3. Elgarisi, M., Frumkin, V., Luria, O. & Bercovici, M. Fabrication of freeform optical components by fluidic shaping. *Optica* **8**, 1501 (2021).

4. *Low-Gravity Fluid Mechanics: Mathematical Theory of Capillary Phenomena*. (Springer, Berlin Heidelberg, 1987).

5. Langbein, D. W. *Capillary Surfaces: Shape-Stability-Dynamics, in Particular under Weightlessness*. (Springer, Berlin ; New York, 2002).

6. Born, M. & Wolf, E. *Principles of Optics*. (Cambridge University Press, Cambridge, United Kingdom, 2019).

7. Africa, C. C. and N. Specifications & Features - Canon XF705 Professional Camcorder. *Canon Central and North Africa* https://en.canon-cna.com/video-cameras/xf705/specifications/.

8. Hale, G. M. & Querry, M. R. Optical Constants of Water in the 200-nm to 200-µm Wavelength Region. *Appl. Opt.* **12**, 555–563 (1973).
